# Sustained elevated levels of C-reactive protein and ferritin in pulmonary tuberculosis patients remaining culture positive upon treatment initiation

**DOI:** 10.1371/journal.pone.0175278

**Published:** 2017-04-06

**Authors:** Pryscila Miranda, Leonardo Gil-Santana, Marina G. Oliveira, Eliene D. D. Mesquita, Elisangela Silva, Anneloek Rauwerdink, Frank Cobelens, Martha M. Oliveira, Bruno B. Andrade, Afrânio Kritski

**Affiliations:** 1 Tuberculosis Academic Program, Medical School, Federal University of Rio de Janeiro, Rio de Janeiro, Brazil; 2 Instituto Gonçalo Moniz, Fundação Oswaldo Cruz, Salvador, Brazil; 3 Multinational Organization Network Sponsoring Translational and Epidemiological Research (MONSTER) Initiative, Fundação José Silveira, Salvador, Brazil; 4 Curso de Medicina, Faculdade de Tecnologia e Ciências (FTC), Salvador, Brazil; 5 Ary Parreira Institute, State Secretary of Health of Rio de Janeiro, Rio de Janeiro, Brazil; 6 Recognize the Biology Laboratory, Center of Bioscience and Biotechnology, State University of North Fluminense Darcy Ribeiro, Rio de Janeiro, Brazil; 7 Rede Brasileira de Pesquisas em Tuberculose (Rede TB), Rio de Janeiro, Brazil; 8 Amsterdam Institute for Global Health and Development, Academic Medical Centre, Amsterdam, The Netherlands; 9 Development Center for Technology on Health (CDTS) Fundação Oswaldo Cruz, Rio de Janeiro, Brazil; 10 Escola Bahiana de Medicina e Saúde Pública, Salvador, Brazil; 11 Universidade Salvador (UNIFACS), Laureate International Universities, Salvador, Brazil; Institut de Pharmacologie et de Biologie Structurale, FRANCE

## Abstract

**Background:**

Clinical trials that evaluate new anti-tubercular drugs and treatment regimens take years to complete due to the slow clearance of *Mycobacterium tuberculosis* infection and the lack of early biomarkers that predict treatment outcomes. Host Inflammation markers have been associated with tuberculosis (TB) pathogenesis. In the present study, we tested if circulating levels of C-reactive protein (CRP) and ferritin reflect mycobacterial loads and inflammation in pulmonary TB (PTB) patients undergoing anti-tuberculous therapy (ATT).

**Methods:**

Prospective measurements of CRP and ferritin, used as readouts of systemic inflammation, were performed in cryopreserved serum samples from 165 Brazilian patients with active PTB initiating ATT. Associations between levels of these laboratory parameters with mycobacterial loads in sputum as well as with sputum conversion at day 60 of ATT were tested.

**Results:**

Circulating levels of both ferritin and CRP gradually decreased over time on ATT. At pre-treatment, concentrations of these parameters were unable to distinguish patients with positive from those with negative acid-fast bacilli (AFB) in sputum cultures. However, patients who remained with positive cultures at day 60 of ATT exhibited heightened levels of these inflammatory markers compared to those with negative cultures at that time point.

**Conclusions:**

CRP and Ferritin levels in serum may be useful to identify patients with positive cultures at day 60 of ATT.

## Introduction

Despite intense investment from both governmental and private sectors, tuberculosis (TB) continues to cause substantial morbidity and mortality in several countries worldwide. A major concern in TB control is the emergence of multidrug-resistant (MDR) and extensively drug-resistant (XDR) TB in various endemic regions [1,2]. In places where there is emergence of drug resistance, TB eradication becomes difficult and the mortality rates documented at 24 and 60 months from diagnosis in these regions are 46% and 73% of patients, respectively [3].

Clinical trials that evaluate new anti-tubercular drugs and treatment regimens take years to complete due to the slow clearance of *Mycobacterium tuberculosis* infection and the lack of early biomarkers that predict treatment outcome [4,5]. Persistence of positive cultures at month 2 of anti-tubercular treatment (ATT) has been proposed as predictor for treatment failure and relapse [6]. There are no well-established host-derived biomarkers that are reliable surrogate markers of successful treatment in large prospective studies. A better understanding of the utility of immune and inflammatory markers as readouts of treatment outcomes can potentially lead to development of innovative prediction tools as well as to establishing targets for host-directed therapies to optimize treatment efficacy.

Biomarkers from the iron pathway, which also serve as acute phase proteins, such as ferritin, could have potential use as treatment response markers beyond just reflecting inflammatory responses [7–9]. Indeed, levels of ferritin, an iron storage protein used as an indicator of iron status and inflammation response, are increased in autoimmune diseases, infections, malignancy and other diseases [10–12]. There is considerable evidence of the contribution of iron availability to mycobacterial growth in vitro as well as in experimental animal models [13,14], but reports on host iron status in response to anti-mycobacterial treatment are scarce. Among pulmonary TB (PTB) patients, high ferritin levels have been reported [15–18], but few studies evaluated if ferritin levels reflect treatment outcomes [7,19–21]. On the other hand, circulating levels of C-reactive protein (CRP), an established biomarker of systemic inflammation, have been described to reflect both TB disease severity and radiographic improvement after 2 months of ATT [22,23]. The present study prospectively examined the association between serum CRP and ferritin levels and *M*. *tuberculosis* bacillary loads in sputum at pre-treatment and after 2 months of ATT in a cohort of treatment-naïve PTB patients from Brazil. The main aim of the study was to test if circulating levels of CRP and ferritin reflect mycobacterial loads and inflammation in PTB patients undergoing ATT. Our hypothesis was that increased circulating levels of these markers would mirror heightened bacterial burden in sputum samples from PTB patients after ATT initiation.

## Materials and methods

### Ethics statement

All clinical investigations were conducted according to the principles expressed in the Declaration of Helsinki. Written informed consent was obtained from each participant at the study enrollment. The study was approved by the Ethics Committee of the University Hospital of Federal University of Rio de Janeiro protocol number: 151/ 05, Ethics committee approval number: 004/ 05 at 28/04/2005). All materials given to the research team were de-identified.

### Study design

We performed a longitudinal cohort study involving 165 patients diagnosed with PTB and admitted to a referral hospital for the treatment of TB in the state of Rio de Janeiro, Brazil (Instituto Estadual Ary Pareiras), between March 2007 and December 2010. Patients that met the inclusion criteria were consecutively admitted over the study period.

Inclusion criteria included confirmatory diagnosis of pulmonary TB performed by sputum culture. First, sputum samples were submitted to microscopic examination of acid-fast bacilli (AFB) in Ziehl-Neelsen stained smears as initial screening. Second, independently of the AFB smear screening, all samples were processed using the Kubica method [24] and inoculated onto Lowenstein-Jensen medium. Mycobacterial loads in smears and cultures were graded following the World Health Organization (WHO) recommendations as negative, scanty, 1+, 2+ or ≥3+. All strains were identified as *M*. *tuberculosis* by culture characteristics and by classical biochemical testing. Drug susceptibility testing was performed for first line drugs with proportion method [25]. There were four patients in the study with the following drug resistance patterns: one with isoniazid mono-resistance, one with streptomycin mono-resistance, and two with combined isoniazid-streptomycin resistance. None was resistant to rifampin.

The HIV-infection was diagnosis by the Determine HIV1/2 Abbot quick^®^ kit following the manufacturer’s instructions. All HIV cases detected in the study participants were caused by HIV-1. The HIV-infected patients recruited were all naïve of antiretroviral therapy.

We excluded subjects aged under 18 and over 60 years, taking anti-tuberculous drugs before admission, with type I diabetes mellitus, renal failure or hemodialysis and peritoneal dialysis blood transfusion, women in pregnancy or lactation period and those whose clinical samples were not subjected to bacteriological or laboratory tests. The baseline characteristics of the study participants are shown in Table 1.

**Table 1:** Characteristic of study population.

Characteristic	N = 165
**Male–no. (%)**	125 (75.7)
**Median Age–years (IQR)**	40 (31–48)
**BMI–kg/m^2^ (IQR)**	17.5 (15.9–19.9)
**Hb–g/dL (IQR)**	11.1 (9.5–12.4)
**Lifestyle habits–no. (%)**	
Chronic Alcoholism	98 (59.4)
Smoking	121 (73.3)
Illicit drug use	47 (28.5)
**Comorbidity–no. (%)**	
Type II diabetes mellitus	5 (3.0)
HIV/AIDS	17 (10.3)
COPD	5 (3.0)
Anemia (Hb < 12.5g/dL)	125 (75.7)

Criterion for anemia in adults was hemoglobin (Hb) value of less than 12.5 g/dL, following standards of the World Health Organization. BMI, body mass index; COPD, chronic obstructive pulmonary disease; IQR, interquartile range.

### Blood collection and Immunoassays

Serum samples were collected at the indicated study timepoints using vacuum blood collection tubes (BD Vacuntainer^®^, Becton Dickinson, Franklin Lakes, NJ). Serum was separated by centrifugation following the manufacturer’s instructions. Samples were aliquoted in cryotubes and stored at -80^°^C until use in the immunoassays. Serum levels of CRP (Ebioscience, San Diego, CA) and ferritin (Abnova, Taipei, Taiwan) were determined using an ELISA-based assay following the manufacturers’ protocols.

### Data analysis

Median and interquartile ranges (IQR) were used as measures of central tendency. Levels of inflammatory markers were compared in the study population. Correlations between the markers were assessed using the Spearman rank test and non-linear curve fit was used to illustrate the overall trend of data distribution in correlation plots. Kruskal-Wallis test with Dunn’s multiple comparisons were utilized for analyses of mycobacterial loads accessed by AFB and culture grade. Median values of biomarkers levels in different groups stratified by AFB and culture status were tested using the Mann-Whitney *U* test. A logistic regression analysis model was employed to test associations between increases of 1 log_10_ in ferritin values measured at day 60 of ATT and *M*. *tuberculosis* culture positivity at that study time point. Further adjustments for age, gender, BMI, HIV infection status and CRP levels were performed. To avoid multicollinearity in our regression model, before performing the logistic regression analysis, we tested the variables for collinearity by perturbing the data using a statistical package in R software (https://cran.r-project.org/web/packages/perturb/index.html). All comparisons were pre-specified. Two-sided p values of <0.05 were considered statistically significant. Statistical analyses were performed using SPSS 20.0 (IBM statistics), Graphpad Prism 7.0 (GraphPad Software, San Diego, CA) and R 3.1.0 (R Foundation, Vienna, Austria).

## Results

We studied a total of 165 PTB patients, with median age of 40 years (IQR: 31–48). The majority of the patients were male (75.7%) and displayed median BMI of 17.5 kg/m^2^ (IQR: 15.9–19.9) (Table 1). Median hemoglobin level was 11.1 g/dL (IQR: 9.5–12.4). In regards to life style habits, 73% were current tobacco smokers, 59.4% had chronic alcoholism according to the CAGE criteria [26], and 28.5% reported illicit drugs use. The most frequent comorbidities were anemia (75%), HIV/AIDS (10%), type II diabetes (3%) and chronic obstructive pulmonary disease (3%) (Table 1).

**Fig 1:**
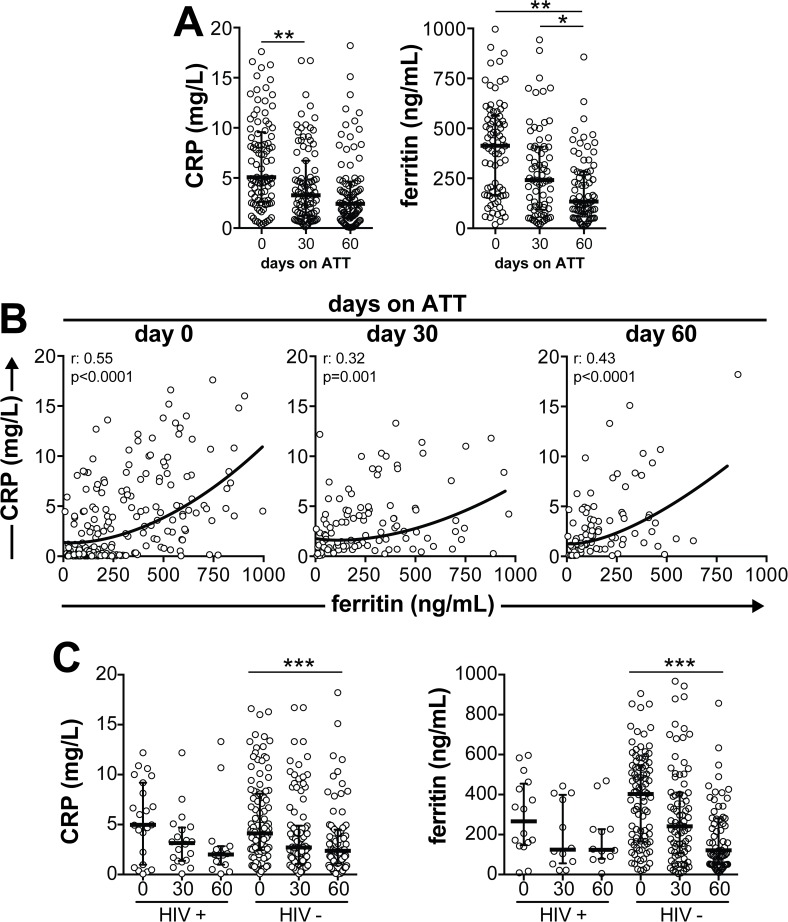
Circulating levels of ferritin, but not CRP, substantially decrease in pulmonary TB patients undergoing anti-tuberculous therapy (ATT). **(A)** Serum concentrations of ferritin and CRP were prospectively measured in a cohort of 165 treatment-naïve patients with culture confirmed pulmonary TB. Lines and whiskers represent median and interquartile values. Data were analyzed using Kruskal Wallis test with Dunn’s multiple comparisons. Statistically significant differences are highlighted. *P<0.05; **P<0.01. **(B)** Serum levels of Ferritin and CRP were tested for correlations at different study time points. Data were analyzed using the Spearman’s rank test. Non-linear curve fit analysis was used to illustrate the trends in data distribution in the correlation plots. **(C)** Concentrations of ferritin and CRP were examined prospectively in patients stratified by HIV infection status.

Circulating levels of both CRP and ferritin at initiation of ATT were negatively correlated with BMI (CRP: r = -0.66, p<0.0001; ferritin: r = -0.29, p<0.0001) and positively correlated with age (CRP: r = 0.54, p<0.0001; ferritin: r = 0.53, p<0.0001). Out of all patients recruited, 37 patients did not return to the follow up visit at day 30 and 32 individuals missed the visit at day 60 of ATT. Twenty-six patients missed both follow up visits and we had only baseline assessment from those. Prospective analyses demonstrated that CRP levels consistently decreased between day 0 and day 30 of treatment, with a trend to further decrease by day 60 (Fig 1A). At day 30 of ATT, ferritin levels presented a trend to decrease compared to pre-treatment values. Furthermore, serum levels of ferritin substantially decreased at day 60 of ATT compared with previous study time points (Fig 1A). Of note, we observed that ferritin and CRP levels were positively correlated not only at pre-treatment (r = 0.55, p<0.0001) but also at day 30 (r = 0.32, p = 0.001) and day 60 (r = 0.43, p<0.0001) of therapy (Fig 1B). Interestingly, levels of both CRP and ferritin gradually decreased in HIV-uninfected patients undergoing anti-TB treatment whereas there were non-statistically significant trends to decrease in in HIV-infected persons (Fig 1C). These findings indicate that although both CRP and ferritin levels substantially decrease upon ATT initiation, patients who remained with high concentrations of CRP simultaneously displayed elevated levels of ferritin. The above results also indicate that some individuals exhibit a residual systemic inflammation at the end of the intensive phase of TB treatment.

**Fig 2:**
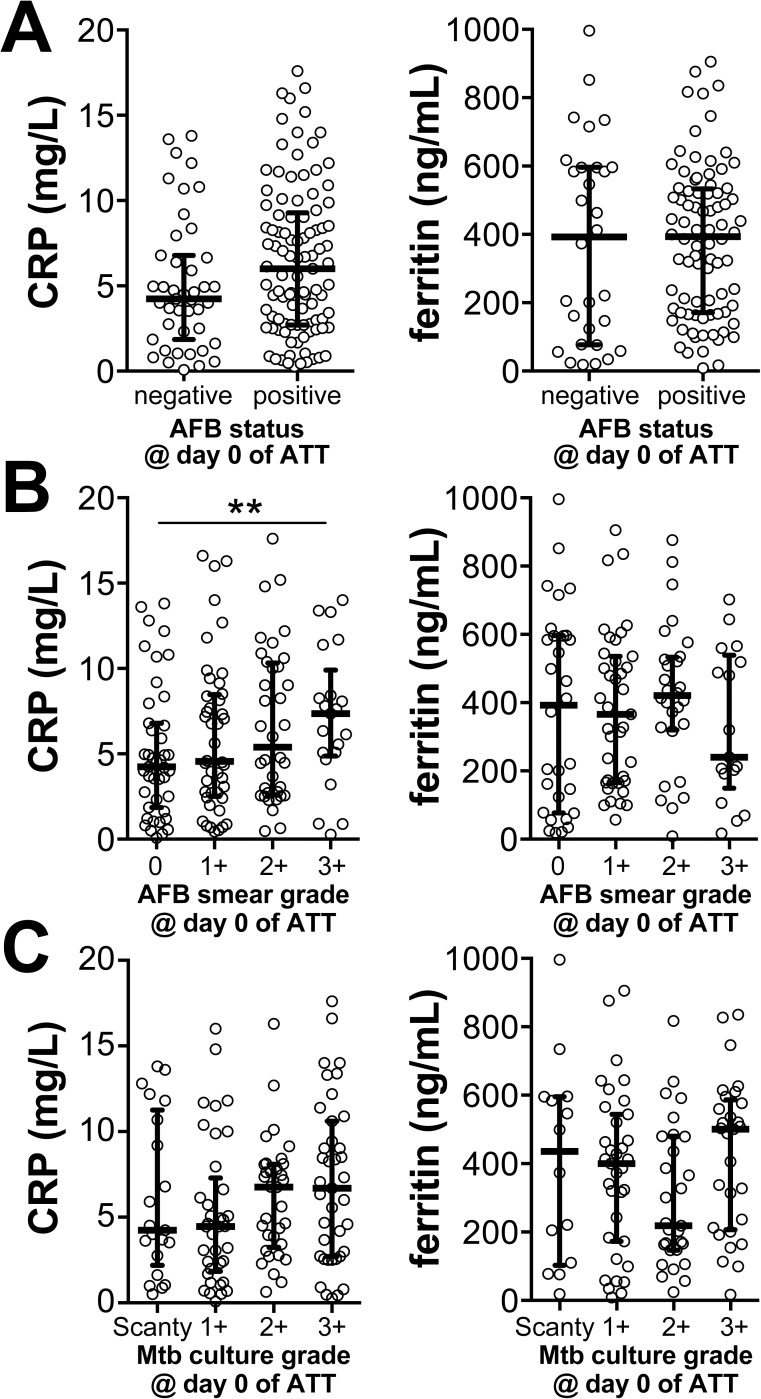
CRP and Ferritin concentrations in serum of treatment-naïve pulmonary TB patients are not significantly influenced by mycobacterial loads in sputum. **(A)** CRP and ferritin levels were compared between pulmonary TB patients with negative or positive identification of acid-fast bacilli (AFB) in sputum smears using the Mann-Whitney test (CRP: p = 0.094, Ferritin: p = 0.830). **(B**) These markers were further compared between groups of TB patients presenting with different sputum smear grades (**p<0.01, AFB ≥3+ vs. 0) or **(C)** those exhibiting different culture grades using Kruskal Wallis test with Dunn’s multiple comparisons. Statistically significant differences are highlighted.

We next examined whether the circulating concentrations of these two markers are associated with mycobacterial loads in sputum in PTB patients before ATT initiation. CRP and ferritin levels were undistinguishable between individuals presenting with positive or negative AFB identification in sputum smears (Fig 2A). Furthermore, we analyzed the variation of this marker according to AFB smear grade and observed that CRP levels were gradually increased following AFB smear grades (linear trend analysis p<0.0001), with the highest values observed in individuals with AFB of ≥3+ (p = 0.0094, compared to AFB = 0; Fig 2B). In this setting, ferritin concentrations presented considerable variability within the different groups and median values did not significantly change following increases in AFB smear grade. In addition, neither CRP or ferritin values could distinguish patients with different bacterial loads in sputum cultures (Fig 2C). These results indicated that CRP, but not ferritin, levels reflect pre-treatment bacterial loads as observed in AFB smear grades in sputum samples in the study population.

**Fig 3:**
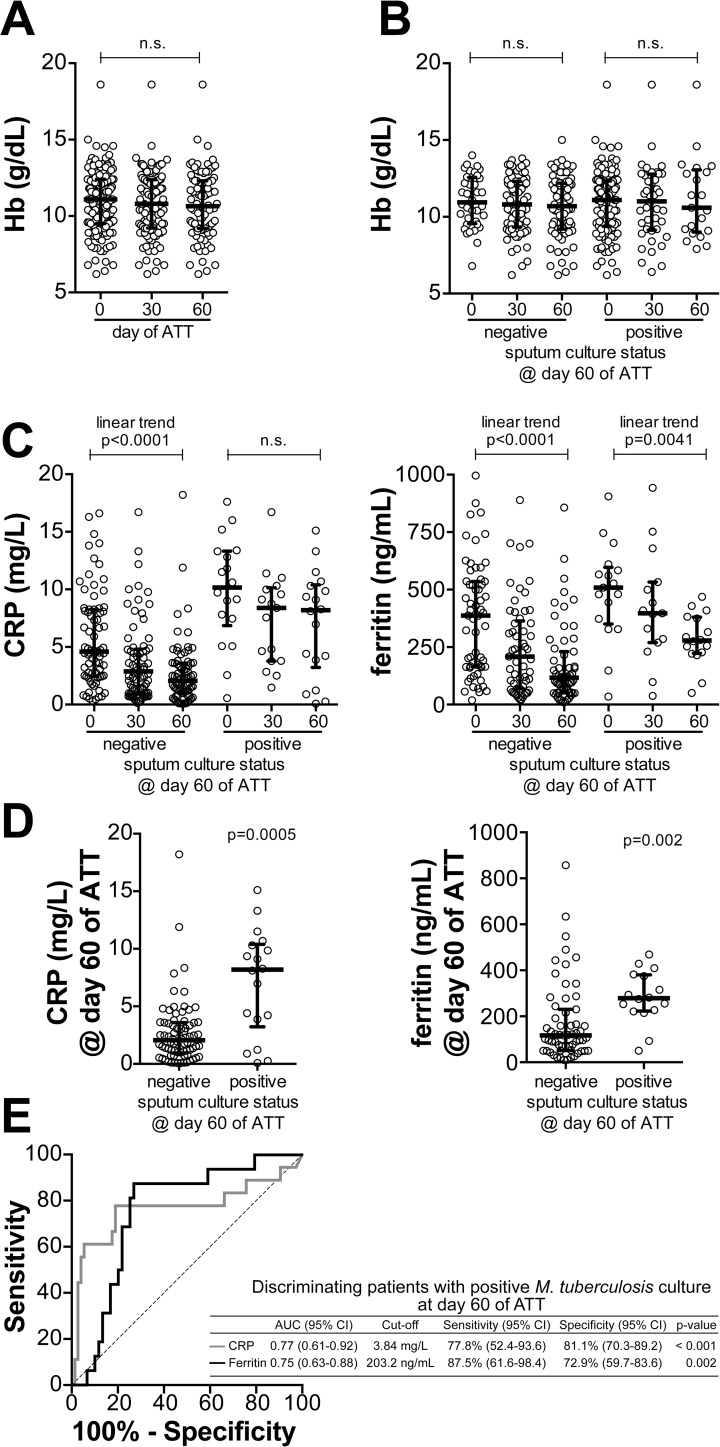
Pulmonary TB patients remaining with culture positive after 60 days of ATT displayed heightened levels of serum ferritin than those who had negative cultures. **(A)** Changes in hemoglobin levels following initiation of ATT are shown. **(B)** Hemoglobin levels upon ATT initiation in TB patients whose sputum cultures became negative at day 60 of ATT and in those who remained culture positive. **(C)** Serum concentrations of CRP and ferritin were compared between the indicated timepoints of ATT in the group of TB patients whose sputum cultures became negative at day 60 of ATT and in that of patients who remained culture positive. In **(A)**, **(B)** and **(C)**, data were compared using Kruskal Wallis test with non-parametric linear trend ad hoc test or Dunn’s multiple comparisons (for CRP). Statistically significant differences are highlighted. **(D)** CRP and Ferritin levels were compared between individuals with culture negative and those with culture positive at day 60 of ATT using the Mann-Whitney *U* test. **(E)** Performance of each marker in identifying patients who had positive cultures at day 60 of ATT was tested using Receiver Operator Characteristics (ROC) curve analysis.

Considering that circulating levels of both CRP and ferritin usually reflect the degree of systemic inflammation, we tested the hypothesis that patients remaining with positive *M*. *tuberculosis* cultures at day 60 of ATT would persist with high levels of these markers, meaning residual inflammation. Of note, hemoglobin levels did not significantly change from pre-ATT to day 30 or 60 of treatment in the entire study population (Fig 3A). In addition, at these time points, hemoglobin levels could not distinguish patients remaining *M*. *tuberculosis* culture positive from those who exhibited negative cultures at day 60 (Fig 3B). These findings suggest that the effects of ATT on hemoglobin levels may require more time to happen, at least in the study population. Nevertheless, in the group of individuals who became culture negative at day 60 of treatment, both CRP and ferritin levels displayed a significant trend to gradually decrease after treatment initiation (Fig 3C). In patients who remained culture positive at day 60, ferritin levels also exhibited a gradual trend to decrease compared to pre-treatment measurements (Fig 3C). Intriguingly, in this group of patients, CRP concentrations significantly decreased by day 30 (p = 0.025), but were further unchanged by day 60 of ATT (Fig 3C). We next compared CRP and ferritin levels measured at day 60 of ATT between these groups of patients diverging in terms of *M*. *tuberculosis* culture conversion. Concentrations of both CRP and ferritin were found to be higher in patients who remained culture positive at day 60 than those who became culture negative (Fig 3D). Receiver Operator Characteristics (ROC) curve analysis revealed that both markers exhibited good performance in distinguishing patients with positive or negative cultures at day 60 of ATT (Fig 3E). In this discriminant analysis, CRP levels displayed higher sensitivity than ferritin concentrations (87.5% vs. 77.8%, respectively) while the latter exhibited increased specificity (81.1% vs. 72.9%) (Fig 3E). Interesting, a logistic regression model revealed that associations between heightened ferritin levels at day 60 and positive cultures at that time point (unadjusted Odds ratio [OR]: 2.05, 95% confidence interval [CI]: 1.15–3.58, p = 0.003) persisted after adjustment for age, gender, BMI, HIV infection status and CRP levels (adjusted OR: 1.54, 95% CI: 1.18–2.32, p = 0.025).

## Discussion

Early biomarkers that predict TB treatment outcome are urgently needed [4,5]. This study investigated potential biomarkers for response to TB treatment. In our investigation, the levels of ferritin and CRP were increased before ATT. We further demonstrated that circulating levels of ferritin, and to a less extent CRP, decreased at day 60 of ATT, as described by others [7,19,20,23]. Interesting, median hemoglobin levels did not significantly change after ATT initiation, suggesting that changes in inflammatory markers induced by treatment may precede the resolution of anemia in the study population. Additional prospective studies are warranted to validate this idea. Jacobs et al [7], evaluating concentrations of 33 biomarkers in 32 pulmonary TB patients, found that the decrease of ferritin levels during anti-TB treatment became significant only by the end of treatment (6 months). Similarly, Minchela et al [20] described a substantial drop in hepcidin concentrations observed only after 2 months of TB treatment, while ferritin levels decreased at earlier stages.

Interestingly, we did not observe a relationship between bacterial loads and serum ferritin levels, suggesting that the mechanisms involved in serum ferritin concentration are independent of the microbial infection burden in sputum. Importantly, mycobacterial loads in sputum samples may not always be a good reflection of total pulmonary bacterial load, especially in immunosuppressed patients. On the other hand, serum ferritin and CRP concentrations appear to be potential biomarkers for positive cultures after 60 days of treatment. Given the known influence of inflammation on plasma ferritin concentrations, Isanaka et al [19] observed that high ferritin levels were associated with culture positive at day 30 of ATT among HIV seronegative patients, after adjusting for CRP values. Our findings showing that elevated ferritin levels at day 60 of ATT were associated with persistent sputum cultures are consistent with this idea.

Higher levels of ferritin before ATT were not observed among smokers, alcoholics and HIV infected subjects as described by others [11,23]. Higher CRP and ferritin levels before ATT were however associated with older age, and low BMI as described by Isanaka et al [19].

Our study had limitations similar to other published studies on TB biomarkers. Our study examined a cohort of patients with active PTB and we did not enroll individuals with latent TB or uninfected persons. Since the main hypothesis of our study was to evaluate effect of ATT on inflammation, comparisons using these control groups are not strictly necessary, although desirable. In addition, because we only had follow-up data until day 60, we were not able assess associations of high ferritin and/or CRP levels with mortality or recurrence/relapse as described previously [19]. As an indicator of iron status and inflammatory response, ferritin values present low performance, but they may be useful in low resource settings where liver biopsy and bone marrow aspiration, the gold standard for the diagnosis of iron overload and deficiency, are not available. Hemoglobin and ferritin values are described to be different in men and women, which may affect the results of this study. The majority of our study population was composed by male individuals, which potentially restricts the inferential power of the results presented here. Additional studies in populations in which female patients are more frequent are necessary to provide definite validation of the findings on prospective evaluation of hemoglobin and ferritin levels. Furthermore, the number of subjects which loss to follow up was high, especially for day 60 of ATT, which was likely caused by the fact that our study was performed in a referral hospital. It is possible that patients who exhibited better clinical response to treatment were discharged from hospital before the end of month 2 of ATT. According to this idea, patients who lost follow up might have had different *M*. *tuberculosis* load and thus this could affect the power to detect treatment outcomes of the present study. We did not have samples available to collect information on other iron status markers, such as hepcidin or transferrin, as well as on additional inflammatory parameters, such as serum amyloid protein or alpha-2-macroglobulin. Thus, a detailed analysis of inflammation and iron status was not performed. Regardless, ferritin and CRP are validated markers of inflammation and our results from a large TB patient cohort clearly demonstrate a link between persistence of culture positivity at day 60 and sustained elevated levels of these parameters. There are advantages of using reliable serum biomarkers to infer persistence of culture positivity upon ATT initiation. Measuring both ferritin and CRP is feasible to be rapidly performed in referral medical centers and at relatively low cost. We thus propose that if the results presented here are validated by future studies with different populations and with different TB epidemiology, CRP and ferritin could be used to monitor treatment response as an adjunct tool together with microbiological approaches. Furthermore, the findings demonstrating a direct association between biomarkers of systemic inflammation and positive culture at day 60 of ATT argues that TB patients with higher risk of treatment failure or relapse persist with a significant degree of inflammatory activity, and such inflammatory profile still requires more detailed characterization.

## Conclusions

We found that ferritin and CRP levels were increased in TB patients starting ATT and decreased during the first 2 months of treatment. While elevated CRP levels reflected higher AFB smear grades, ferritin levels were not associated by mycobacterial load. This observation leads us to speculate that *M*. *tuberculosis* driven inflammatory tissue damage may trigger robust induction of CRP production whereas ferritin may be induced indirectly by activation of other inflammatory pathways, which have yet to be delineated. Finally, ferritin showed to be a promising TB biomarker in predicting anti-tuberculosis treatment outcome at 60 days of anti-tuberculous therapy.

## Supporting information

S1 File Individual data points behind means, medians and variance measures presented in the results. (PDF)

## References

[1] World Health Organization (2015) Global tuberculosis report 2015. http://apps.who.int/iris/bitstream/10665/191102/1/9789241565059_eng.pdf?ua=1 (assessed on 11/12/2016)

[2] World Health Organization (2015) Fact sheets on tuberculosis; Multidrug-Resistant Tuberculosis (MDR-TB): 2015 Update. http://www.who.int/tb/challenges/mdr/mdr_tb_factsheet.pdf (assessed on 11/12/2016)

[3] Petersen E, Chen LH (2015) Reflections on travel-associated infections in Europe. Lancet Infect Dis 15: 6–8. doi: 10.1016/S1473-3099(14)71055-2 25477023 10.1016/S1473-3099(14)71055-2 PMC7128710

[4] Clifford V, Zufferey C, Street A, Denholm J, Tebruegge M, Curtis N (2015) Cytokines for monitoring anti-tuberculous therapy: A systematic review. Tuberculosis (Edinb) 95: 217–228. 25797612 10.1016/j.tube.2015.01.003

[5] Walzl G, Haks MC, Joosten SA, Kleynhans L, Ronacher K, Ottenhoff TH (2014) Clinical immunology and multiplex biomarkers of human tuberculosis. Cold Spring Harb Perspect Med 5. 10.1101/cshperspect.a018515 PMC4382732 25475107

[6] Wallis RS, Maeurer M, Mwaba P, Chakaya J, Rustomjee R, Migliori GB, et al (2016) Tuberculosis—advances in development of new drugs, treatment regimens, host-directed therapies, and biomarkers. Lancet Infect Dis 16: e34–46. doi: 10.1016/S1473-3099(16)00070-0 27036358 10.1016/S1473-3099(16)00070-0

[7] Jacobs R, Tshehla E, Malherbe S, Kriel M, Loxton AG, Stanley K, et al (2016) Host biomarkers detected in saliva show promise as markers for the diagnosis of pulmonary tuberculosis disease and monitoring of the response to tuberculosis treatment. Cytokine 81: 50–56. doi: 10.1016/j.cyto.2016.02.004 26878648 10.1016/j.cyto.2016.02.004

[8] Kuznetsov IA, Rasulov MM, Iskakova JT (2013) Iron-containing proteins lactoferrin and ferritin in biological media of patients with pulmonary tuberculosis. Bull Exp Biol Med 154: 618–621. 23658882 10.1007/s10517-013-2013-8

[9] Minchella PA, Donkor S, Owolabi O, Sutherland JS, McDermid JM (2015) Complex anemia in tuberculosis: the need to consider causes and timing when designing interventions. Clin Infect Dis 60: 764–772. doi: 10.1093/cid/ciu945 25428413 10.1093/cid/ciu945

[10] Bouharras El Idrissi H, Molina Lopez J, Perez Moreno I, Florea DI, Lobo Tamer G, Herrera-Quintana L, et al (2015) Imbalances in Protein Metabolism in Critical Care Patient with Systemic Inflammatory Response Syndrome at Admission in Intensive Care Unit. Nutr Hosp 32: 2848–2854. doi: 10.3305/nh.2015.32.6.9827 26667743 10.3305/nh.2015.32.6.9827

[11] Koethe JR, Blevins M, Nyirenda C, Kabagambe EK, Shepherd BE, Wester CW, et al (2011) Nutrition and inflammation serum biomarkers are associated with 12-week mortality among malnourished adults initiating antiretroviral therapy in Zambia. J Int AIDS Soc 14: 19 doi: 10.1186/1758-2652-14-19 21477359 10.1186/1758-2652-14-19 PMC3094357

[12] Tacke F, Nuraldeen R, Koch A, Strathmann K, Hutschenreuter G, Trautwein C, et al (2016) Iron Parameters Determine the Prognosis of Critically Ill Patients. Crit Care Med 44: 1049–1058. doi: 10.1097/CCM.0000000000001607 26934143 10.1097/CCM.0000000000001607

[13] Reddy PV, Puri RV, Khera A, Tyagi AK (2012) Iron storage proteins are essential for the survival and pathogenesis of Mycobacterium tuberculosis in THP-1 macrophages and the guinea pig model of infection. J Bacteriol 194: 567–575. doi: 10.1128/JB.05553-11 22101841 10.1128/JB.05553-11 PMC3264086

[14] Tanaka M, Kanamori H, Matsumoto K, Tachibana T, Numata A, Ohashi K, et al (2015) Clinical significance of pretransplant serum ferritin on the outcome of allogeneic hematopoietic SCT: a prospective cohort study by the Kanto Study Group for Cell Therapy. Bone Marrow Transplant 50: 727–733. doi: 10.1038/bmt.2015.17 25730191 10.1038/bmt.2015.17

[15] Friis H, Range N, Braendgaard Kristensen C, Kaestel P, Changalucha J, Malenganisho W, et al (2009) Acute- phase response and iron status markers among pulmonary tuberculosis patients: a cross-sectional study in Mwanza, Tanzania. Br J Nutr 102: 310–317. doi: 10.1017/S0007114508162122 19175946 10.1017/S0007114508162122

[16] McDermid JM, Hennig BJ, van der Sande M, Hill AV, Whittle HC, Jaye A, et al (2013) Host iron redistribution as a risk factor for incident tuberculosis in HIV infection: an 11-year retrospective cohort study. BMC Infect Dis 13: 48 doi: 10.1186/1471-2334-13-48 23360117 10.1186/1471-2334-13-48 PMC3568026

[17] Oliveira MG, Delogo KN, Oliveira HM, Ruffino-Netto A, Kritski AL, Oliveira MM (2014) Anemia in hospitalized patients with pulmonary tuberculosis. J Bras Pneumol 40: 403–410. doi: 10.1590/S1806-37132014000400008 25210963 10.1590/S1806-37132014000400008 PMC4201171

[18] Subbian S, Pandey R, Soteropoulos P, Rodriguez GM (2015) Vaccination with an Attenuated Ferritin Mutant Protects Mice against Virulent Mycobacterium tuberculosis. J Immunol Res 2015: 385402. 10.1155/2015/385402 PMC4539171 26339659

[19] Isanaka S, Aboud S, Mugusi F, Bosch RJ, Willett WC, Spiegelman D, et al (2012) Iron status predicts treatment failure and mortality in tuberculosis patients: a prospective cohort study from Dar es Salaam, Tanzania. PLoS One 7: e37350 doi: 10.1371/journal.pone.0037350 22606361 10.1371/journal.pone.0037350 PMC3350480

[20] Minchella PA, Donkor S, McDermid JM, Sutherland JS (2015) Iron homeostasis and progression to pulmonary tuberculosis disease among household contacts. Tuberculosis (Edinb) 95: 288–293. 25764944 10.1016/j.tube.2015.02.042

[21] Sivakolundu S, Mannela UD, Jain S, Srikantam A, Peri S, Pandey SD, et al (2013) Serum iron profile and ELISA-based detection of antibodies against the iron-regulated protein HupB of Mycobacterium tuberculosis in TB patients and household contacts in Hyderabad (Andhra Pradesh), India. Trans R Soc Trop Med Hyg 107: 43–50. doi: 10.1093/trstmh/trs005 23222944 10.1093/trstmh/trs005

[22] Mayer-Barber KD, Andrade BB, Oland SD, Amaral EP, Barber DL, Gonzales J, et al (2014) Host-directed therapy of tuberculosis based on interleukin-1 and type I interferon crosstalk. Nature 511: 99–103. doi: 10.1038/nature13489 24990750 10.1038/nature13489 PMC4809146

[23] Plit ML, Theron AJ, Fickl H, van Rensburg CE, Pendel S, Anderson R (1998) Influence of antimicrobial chemotherapy and smoking status on the plasma concentrations of vitamin C, vitamin E, beta-carotene, acute phase reactants, iron and lipid peroxides in patients with pulmonary tuberculosis. Int J Tuberc Lung Dis 2: 590–596. 9661828

[24] Kubica GP, Dye WE, Cohn ML, Middlebrook G (1963) Sputum digestion and decontamination with N-acetyl-L-cysteine-sodium hydroxide for culture of mycobacteria. Am Rev Respir Dis 87: 775–779. doi: 10.1164/arrd.1963.87.5.775 13927224 10.1164/arrd.1963.87.5.775

[25] World Health Organization (1998) Laboratory Services in Tuberculosis Control, Part II; WHO, editor. Geneva: World Health Organization.

[26] Mayfield DG, McLead G, Hall P (1974) The CAGE questionnaire validation of a new alcoholism screening instrument. Am J Psychiatry 131: 1121–1123. doi: 10.1176/ajp.131.10.1121 4416585 10.1176/ajp.131.10.1121

