# Supplementary material for: Sustained elevated levels of C-reactive protein and ferritin in pulmonary tuberculosis patients remaining culture positive upon treatment initiation
Source: PLoS One. 2017 Apr 6;12(4):e0175278. doi: 10.1371/journal.pone.0175278 (PMC5383283; doi:10.1371/journal.pone.0175278)
Supplement: S1 File — (PDF) [file pone.0175278.s001.pdf]

STUDY ID	GENDER	BMI	AGE	HEMOGLOBIN	Chronic Alcoholism	Illicit drug use	Smoking	Ferritin Day 0	CRP day 0	positive BAAR day 60	positive Culture day 60
11	Male	16.93594104	44	12.10	yes	no	no	544.20	2.40		
13	Male	22.63	44	14.00	no	no	yes	142.50	0.38		
14	Male	17.42204267	29	6.80	no	yes	yes	373.10	12.20		
15	Male	14.94646044	31	12.10	yes	no	yes	205.10	0.99		
16	Male	18.11	22	13.10	no	no	no	452.50	1.75		
17	Female	19.64	22	13.00	no	no	no	132.50	2.42		
18	Female	22.12041279	46	10.40	no	no	yes	735.00	10.70		
19	Female	21.10301769	51	11.60	yes	no	yes	160.00	4.70		
20	Male	13.40417824	50	9.70	yes	yes	yes	537.30	7.13		
21	Male	14.67128028	43	11.60	no	no	yes	639.60	8.10		
22	Male	17.01	23	15.00	no	no	no	323.10	1.99		
23	Female	20.52029136	24	10.80	no	no	yes	110.70	0.88		
24	Male	17.43753718	44	6.40	yes	yes	yes	500.60	9.43		
25	Male	20.28680041	39	11.90	yes	no	yes	138.60	2.78		
26	Female	14.16329019	34	10.40	no	no	no	54.10	3.20		
27	Male	16.53	30	12.80	yes	yes	yes	566.10	5.07	positive	positive
29	Male	14.56815817	38	9.60	yes	no	yes	876.40	14.80		
30	Male	16.6644132	42	9.20	yes	no	yes	702.70	5.07	positive	positive
31	Female	16.43068684	40	10.40	yes	no	yes	237.20	2.72		
32	Male	18.67	57	13.30	yes	no	yes	1,059.90	16.30		
33	Male	20.42835711	50	9.70	yes	no	no	826.90	8.46		
34	Male	19.45311597	31	11.40	no	yes	no	480.00	7.80	positive	positive
35	Male	19.72494835	37	12.10	yes	yes	yes	168.00	2.78		
36	Female	13.34	44	12.90	no	no	no	164.60	0.44		
37	Female	22.83287935	32	12.20	no	yes	yes	91.00	2.29		
38	Male	12.36259147	41	8.50	no	no	no	203.10	7.62		
39	Female	13.27251808	41	7.70	no	no	yes	113.80	6.70		
40	Male	not measured	25	10.60	yes	yes	yes	905.80	16.00	positive	positive
41	Male	26.53769841	36	10.80	yes	no	yes	742.10	3.56		
42	Male	24.35	50	13.80	no	no	no	467.60	1.69		
43	Male	20.64886899	23	11.50	no	no	no	162.20	1.86		
44	Male	17.88797062	20	12.20	yes	yes	yes	626.40	4.59		
45	Male	not measured	34	9.50	yes	no	no	1,025.20	4.33		
46	Male	22.35	22	13.80	no	no	no	413.80	1.04		
47	Male	20.12046649	52	7.70	yes	yes	yes	835.50	7.33		
48	Female	19.59	50	13.40	no	no	no	184.00	0.71	positive	
51	Male	21.5450908	64	12.40	yes	no	yes	218.50	3.05		
52	Female	16.44666581	47	8.90	yes	no	no	242.60	1.17		
53	Male	not measured	23	9.60	yes	yes	yes	463.00	4.94		
55	Male	21.63	35	12.70	yes	no	no	201.00	2.51		
57	Male	22.32142857	43	12.20	yes	no	yes	76.00	0.50		
58	Male	16.00	54	not measured	yes	no	yes	386.50	7.68		
59	Male	15.96	43	nor measured	no	no	yes	147.50	9.73		positive
60	Female	13.24	41	not measured	no	no	yes	546.40	3.62		
61	Male	15.25961157	53	9.40	yes	no	yes	610.10	2.56	positive	positive
62	Male	16.30	31	14.60	yes	no	no	479.60	7.46		positive
63	Male	19.61950059	31	11.50	yes	yes	yes	405.80	2.99		
64	Female	17.68978885	46	9.20	no	no	no	220.40	3.99		
65	Male	15.75896631	37	9.30	no	yes	yes	326.30			
67	Male	21.62853844	30	8.50	yes	yes	yes	1,500.00	2.32		
69	Male	15.44	51	12.90	yes	no	yes	413.00	8.26		
72	Male	17.8200692	39	9.20	yes	no	yes	164.00	12.70	positive	
73	Male	17.56259642	26	7.90	yes	yes	no	327.80	9.15		positive
74	Male	16.39399676	27	8.30	yes	no	no	853.50	4.02		
75	Female	17.37286687	36	11.70	yes	yes	yes	146.70	4.93		
76	Female	16.64932362	34	8.10	no	no	no	444.70	11.80	positive	positive
79	Male	22.01814144	53	12.40	yes	no	yes	34.20	0.55		positive
80	Male	21.04855372	43	10.30	no	no	yes	106.20	8.42		
82	Male	18.96192742	48	12.30	no	no	no	317.20	6.63		
85	Male	18.79	25	12.50	no	yes	yes	77.60	1.04		
86	Male	16.97959184	45	6.80	yes	yes	yes	192.20	6.34	positive	
88	Male	16.09904819	50	9.30	yes	no	yes	1,167.90	0.80		
89	Male	16.91723323	21	11.60	no	yes	yes	412.20	4.63		
90	Female	15.76505563	33	10.60	yes	yes	yes	292.00	6.49		
91	Male	16.97959184	34	10.50	no	no	no	596.00	4.19		
92	Female	not measured	42	12.90	no	no	yes	577.00	12.20		
93	Female	19.27	48	13.50	no	no	no	387.00	4.48		
94	Male	not measured	49	8.70	no	no	no	644.00	11.70		
95	Male	21.38	45	12.90	yes	no	yes	488.00	0.91	positive	
96	Female	15.61472715	20	10.50	no	no	yes	57.00	3.87		
97	Female	15.20381328	30	6.20	yes	yes	yes	341.00	4.86		
98	Male	15.625	43	8.70	yes	no	no	439.00	3.08		
99	Male	18.81892431	51	9.10	yes	no	yes	499.00	13.80		
100	Male	22.72	48	13.50	no	no	yes	338.00	6.00		
101	Male	15.50	40	12.80	no	no	yes	301.00	5.62		
102	Female	17.56965112	32	11.40	no	no	no	17.00	0.28		
104	Female	20.07774787	41	10.50	no	no	yes	521.00	2.51		
105	Male	16.81666607	47	9.90	yes	no	yes	584.00	12.80		positive
106	Male	15.88317212	43	8.70	no	no	yes	584.00	0.53		
107	Female	12.30662397	21	8.70	no	yes	yes	560.00	13.30	positive	positive
108	Male	not measured	41	12.30	yes	no	no	166.00	6.77		
109	Female	not measured	36	11.40	yes	yes	yes	328.00	10.60		positive
110	Male	20.69012688	36	9.90	no	yes	yes	606.00	2.70		
111	Male	20.2020202	43	9.30	yes	yes	yes	485.00	4.36		
113	Female	14.09714212	17	7.90	yes	yes	yes	121.00	3.06		
114	Male	15.49586777	49	9.10	no	yes	yes	428.00	10.00		
115	Female	not measured	29	11.60	yes	no	yes	584.00	4.97		
116	Male	16.43598616	47	8.40	yes	no	yes	372.80	11.50	positive	positive
117	Male	18.30192456	52	11.40	yes	no	yes	missing	6.38		
118	Male	17.92470156	51	12.30	yes	no	yes	missing	6.65		
119	Male	18.50191832	27	9.90	yes	yes	yes	399.70	2.32		
120	Male	17.17	31	13.40	yes	yes	yes	535.00	0.64		
121	Female	18.78372348	35	7.10	no	no	yes	592.00	1.68		
122	Male	22.49858277	20	12.40	no	yes	yes	21.60	0.08		
123	Female	21.82644326	46	11.60	no	no	yes	missing	6.79		
124	Female	19.34585419	31	12.40	yes	no	yes	missing	0.47		
126	Male	15.93	48	13.20	yes	no	no	123.50	8.36		
128	Male	16.97	50	18.60	yes	no	yes	508.40	9.03	positive	positive
129	Male	21.2585034	31	10.40	no	no	yes	missing	3.69		
130	Male	21.77	60	13.40	no	no	yes	1,027.20	13.40		positive
131	Male	not measured	49	13.30	no	no	yes	missing	4.95		
132	Male	18.25550268	48	10.80	no	no	yes	99.50	4.46	positive	
133	Male	18.81118647	47	8.90	yes	no	yes	996.20	4.50		
134	Male	14.42752721	56	11.70	yes	no	yes	469.50	3.60		
136	Female	17.06065523	39	9.20	no	no	yes	missing	0.30		
137	Male	17.16262976	49	9.60	yes	no	yes	852.60	10.80		
138	Male	14.45637119	42	10.70	yes	no	yes	715.40	4.74		
139	Male	16.51234568	39	11.10	yes	yes	yes	1,384.70	8.15		
140	Female	20.45	30	13.20	yes	yes	yes	596.80	4.00		
141	Female	26.07897153	48	11.80	yes	no	yes	24.30	1.20		
143	Male	17.28925916	25	11.80	yes	no	yes	226.00	3.42		
149	Male	17.8925737	31	8.90	no	no	no	519.00	8.26		
152	Male	21.86851211	52	11.60	yes	no	no	missing	8.00	positive	
154	Male	17.14	39	12.60	no	no	yes	664.00	4.32		
155	Male	17.92800454	48	9.10	yes	no	yes	526.80	15.20	positive	positive
156	Male	24.59	25	12.90	yes	yes	yes	172.80	2.24		
157	Male	20.45073698	50	9.00	yes	no	yes	1,467.40	2.76		
158	Male	16.18443652	53	12.00	yes	no	yes	212.50	4.68		
159	Male	not measured	51	11.30	yes	no	yes	missing	2.44		
160	Male	17.78628118	41	12.30	yes	no	no	missing	3.65		
161	Male	23.81474497	44	9.50	yes	no	yes	missing	10.90		
162	Male	17.47471743	60	9.70	yes	no	yes	missing	10.40		
163	Female	14.27721594	44	10.10	no	no	no	8.80	4.46		
164	Male	17.90905142	33	12.30	yes	yes	yes	172.40	0.70		
169	Male	not measured	41	7.90	no	no	no	69.80	8.08		
173	Female	18.02595738	31	11.80	yes	yes	yes	56.30	4.01		
174	Male	not measured	30	9.80	no	yes	yes	817.00	6.76		
175	Male	13.96638712	53	8.90	yes	no	yes	435.60	10.10		
176	Female	16.89906348	49	11.60	no	no	no	320.40	6.14		
177	Male	18.88558884	32	10.50	no	no	no	2,152.00	2.52		
178	Male	16.82422689	56	9.20	yes	no	yes	206.50	7.36		
179	Male	19.76	25	13.30	no	yes	yes	221.30	13.60		
180	Male	16.31633715	52	10.50	yes	no	yes	617.60	7.96		
181	Male	18.9807492	45	10.10	yes	no	yes	19.00	5.91		
182	Male	17.21	32	13.20	no	no	yes	154.50	3.67		
183	Male	17.63365688	44	10.40	no	yes	yes	746.10	17.60		positive
184	Female	17.99816345	20	10.20	no	no	no	26.90	0.86		
185	Male	not measured	40	9.50	yes	no	yes	595.40	1.63		
186	Male	23.05456246	38	11.30	no	no	yes	521.20	9.89		
187	Male	18.82774473	36	12.10	yes	yes	yes	1,394.00	4.24		
188	Female	25.58844889	51	11.10	no	no	yes	246.50	3.91		
189	Male	17.63	29	13.70	yes	yes	yes	314.20	8.38		
190	Male	15.23055915	40	10.40	yes	no	yes	536.00	16.60		
191	Male	22.98143018	38	12.20	no	yes	yes	58.50	1.19		
192	Male	13.30485627	19	10.20	no	yes	yes	104.40	4.51		
193	Male	16.08734231	40	7.00	yes	no	yes	614.80	14.00		
194	Male	14.70536746	20	8.90	no	yes	yes	99.70	8.51		
195	Male	not measured	30	13.40	no	no	yes	3,598.00	3.53		
197	Male	18.03577294	30	11.50	yes	yes	no	missing	11.80		
198	Male	11.90439852	23	11.10	yes	no	no	missing	5.55		
199	Male	16.18427491	37	8.00	yes	no	yes	missing	2.70		
200	Male	16.41	33	12.70	no	no	no	missing	9.20		
201	Male	20.51	59	12.90	yes	no	yes	missing	11.30		
203	Male	16.09	51	12.80	yes	no	no	missing	2.56		
204	Female	17.49923249	32	9.60	yes	no	no	missing	11.40		
206	Male	23.95122659	58	11.10	yes	no	yes	1,351.00	7.05		
207	Male	23.41	47	14.60	yes	yes	yes	503.00	0.79		
208	Male	18.55368882	39	11.10	yes	no	yes	366.00	3.96		
209	Male	16.45600644	28	8.00	yes	no	no	4,111.00	14.00		
210	Male	19.01	58	13.10	yes	no	yes	1,288.00	4.64		
211	Male	14.69	45	14.50	yes	no	yes	1,458.00	9.02		
212	Female	17.24207879	27	7.90	yes	yes	yes	240.00	0.89		
213	Female	18.25435658	29	10.10	no	no	no	171.00	7.17		
328	Male	19.9806208	55	11.00	yes	no	yes	642.00	5.72		
329	Male	not measured	47	6.90	yes	yes	yes	812.00	4.77		
